# Self-perceived competence of dental students regarding the management of orofacial pain: a cross-sectional study

**DOI:** 10.1186/s12903-021-01852-1

**Published:** 2021-09-27

**Authors:** Muhammad Taqi, Asaad Javed Mirza, Maaz Asaad Javed, Shama Asghar, Maria Khadija, Syed Ali Raza

**Affiliations:** 1 Department of Community Dentistry, Dow Dental College, Dow University of Health Sciences, Mission Rd, New Labour Colony Nanakwara, Karachi City, Sindh, 74200 Pakistan; 2 Department of Operative Dentistry, College of Dentistry, Baqai Medical University, Karachi, Pakistan; 3 Department of Periodontology, College of Dentistry, Baqai Medical University, Karachi, Pakistan; 4 Department of Operative Dentistry, College of Dentistry, Bahria University Medical and Dental College, Karachi, Pakistan; 5 Department of Community Dentistry, Jinnah Medical and Dental College, Karachi, Pakistan; 6 Department of Community Dentistry, Sir Syed College of Medical Sciences for Girls, Karachi, Pakistan

**Keywords:** Facial pain, Dental education, Competence, Temporomandibular joint disorder, Primary headaches, Neuralgia

## Abstract

**Background:**

There is limited data on Pakistani dental students perceived competence in managing orofacial pain (OFP). This study aims to evaluate dental students self-perceived competence regarding the management of orofacial pain.

**Methods:**

This cross-sectional study was conducted in Karachi at randomly selected two public and four private dental schools. This survey was conducted online from November 2020 to December 2020 in six dental schools. A questionnaire link was sent to the 475 students. A chi-square test and independent-sample t-test were conducted to assess the frequency distribution and compare mean scores of knowledge, diagnosis, and management parameters.

**Results:**

Of the 475 students, 280 students filled the online survey leaving a response rate of 59%. A significant number of fourth-year students, 65 (51%, *p* = 0.005), feels knowledgeable regarding neuropathic pain compared to third-year students. The majority of the fourth-year students, 100 (78%, *p* = 0.010), feel comfortable managing intraoral pain. Almost all the students reported thinking that they need more knowledge related to five types of OFP. The fourth-year students had high mean scores related to knowledge, comfort in diagnosing and managing OFP categories.

**Conclusion:**

This study found that dental students perceived competence regarding orofacial pain management varies in relation to specific categories, being lowest for psychogenic pain.

## Background

Pain is the most common health problem. Adverse effects of pain range from management costs, loss of work, reduced productivity, and reduced quality of life [1]. The head, neck, and face pain is defined as orofacial pain (OFP) [2]. The OFP has five different categories: TMD-related pain, intraoral pain, psychogenic pain, neuropathic pain, and primary headaches [3].

The most common cause of orofacial pain is of odontogenic origin and comes under the domain of dental medicine and should not be a diagnostic-therapeutic challenge in itself. On the other hand, non-odontogenic causes of orofacial pain include musculoskeletal, neuropathological diseases, temporomandibular disorders (TMD), neuralgias, ENT diseases, tumours, and neurovascular pain or psychiatric diseases [4, 5]. These conditions usually present overlapping signs and symptoms and present a diagnostic dilemma for the clinician who usually treats the patient for dental pain [4, 5]. Arriving at the correct diagnosis is of crucial importance to guide the delivery of appropriate treatment. Initiating treatment without sufficient attention to the complex mechanism of pain may result in unnecessary treatment without eliminating the problem [4, 5]. In Pakistan, no national data exist regarding the prevalence of orofacial pain. However, studies on the local population reported a 30% prevalence of OFP in 11–14-year-old [5]. Similarly, 43% prevalence of OFP was reported among patients visiting the dental hospitals in Karachi [6].

The most apparent purpose of dental education is to build a future professional who can operate efficiently as a key element of the health care system. In recent years, health professional education in Pakistan has transformed; it is now competency-based, providing a significant framework for curriculum designing and identifying the limitations of delivering these curricula. Competency is an intricate ability essential for the dentist to begin dental practice. It is not limited to executing a few dental procedures; rather, it includes knowledge, experience, critical thinking, problem-solving skills, professionalism, technical and procedural skills [7]. All the components mentioned above are required in combination to provide holistic patient care [8].

A cross-sectional study mentioned that Iranian general dentists did not have enough knowledge about chronic orofacial pain, especially in the treatment field [9]. Similarly, a cross-sectional study reports that Indian post-graduate students have little confidence in managing temporomandibular joint disorders [10]. The dental professional's inability to diagnose OFP will expose patients to unnecessary treatment and affect their finances and routine life.

So far, there is a lack of data related to Pakistani dental students perceived competence regarding the management of OFP. Therefore, it is essential to be clear about the Pakistani dental graduate's qualities to ensure that our graduates are safe dental practitioners and can perform competently. Hence, this study aims to evaluate dental students perceived competence regarding the management of orofacial pain. We hypothesise that fourth-year dental students will report more competency than third-year students related to the management of OFP categories.

## Methods

### Study design and settings

This descriptive quantitative cross-sectional study was conducted at randomly selected two public and four private dental schools of Karachi using the lottery method. All third- and fourth-year students of six dental schools were invited to participate in this survey. A questionnaire link was sent to the 475 students of six dental schools. The survey was conducted online from November 2020 to December 2020, when the clinical training was almost completed.

The survey questionnaire was prepared online using Google Docs, and a link was sent to the study participants using WhatsApp manufactured by WhatsApp Inc. and emails. The reminder emails and WhatsApp messages were sent to the participants to fill the Google survey form after the initial invitation to follow up on the audience. The survey was anonymous, and the participants did not get any compensation. The participants were able to fill the google form only once and could not see the responses from the other participants.

### Educational framework & dental curriculum

In Pakistan, four years of study is required to graduate from dental school. In the preclinical period (year one and year two), education remains theoretical. The dental curriculum in Pakistan was prepared by the curriculum revision committee of the Higher Education Commission, was duly approved by Pakistan Medical Commission- the statutory body for monitoring medical and dental education and health delivery in the country & is circulated for implementation by all the affiliated institutions. All the dental colleges and universities follow this curriculum in Pakistan to get registration of the commission for dental practitioners [11].

As per the dental curriculum, classes directly related to pain management occur during the third and fourth years. In the third year, the lectures are focussed on the following topics' disorders of the temporomandibular joint: clinical features, diagnosis and treatment, clinical features, diagnosis, and treatment of dentoalveolar pain, neurological pains, psychosomatic pain. In the fourth year, courses involve topics of intraoral / pulpal pain management [11].

### Study instrument

The competency item OFP was identified from the first national document on dental graduates' professional competency [12]. The perceived competency (Assessing orofacial pain) was assessed in three parameters knowledge, diagnosis, and management. The questionnaire was in English and adopted from the previous study [13] with the corresponding author permission. The questionnaire was comprised of different areas of dental graduates perceived level of competency regarding orofacial pain.

The survey questionnaire consisted of 5 sections. The first section contains the cover letter explaining the project purpose, assuring them of confidentiality of their responses, and the procedure of answering the questions. Section two was about demographic information and the student's familiarity regarding types of orofacial pain. Section three includes the question on the perceived level of competence regarding managing different types of OFP. The last section asked if they needed more knowledge regarding the management of OFP. A five-point Likert scale was used for the competence questions. Table 1 shows the summary of close-ended survey questions and the responses.

**Table 1:** Summary of questions about the self-perceived knowledge, comfort in diagnosis and managing different types of Orofacial pain

Measure	Question	Response
Knowledge	What is your degree of knowledge related to pain associated with temporomandibular joint disorder?	No knowledge Limited knowledge Not sure Knowledgeable Extremely knowledgeable
	What is your degree of knowledge related to neuropathic pain?	
	What is your degree of knowledge related to intraoral pain?	
	What is your degree of knowledge related to psychogenic pain?	
	What is your degree of knowledge related to primary headaches?	
Diagnosis	How comfortable do you feel diagnosing patients with pain associated with temporomandibular joint disorder?	Very uncomfortable Uncomfortable Neither Comfortable Very comfortable
	How comfortable do you feel diagnosing patients with primary headaches?	
	How comfortable do you feel diagnosing patients with neuropathic pain?	
	How comfortable do you feel diagnosing patients with intraoral pain?	
	How comfortable do you feel diagnosing patients with psychogenic pain?	
Management	How comfortable do you feel managing patients with temporomandibular joint pain?	Very uncomfortable Uncomfortable Neither Comfortable Very comfortable
	How comfortable do you feel managing patients with primary headache pain?	
	How comfortable do you feel managing patients with neuropathic pain?	
	How comfortable do you feel managing patients with intraoral pain?	
	How comfortable do you feel managing patients with psychogenic pain?	
More knowledge	Do you think you need more knowledge related to TMD related pain?	Strongly disagree Disagree Not sure Agree Strongly agree
	Do you think you need more knowledge related to primary headaches?	
	Do you think you need more knowledge related to neuropathic pain?	
	Do you think you need more knowledge related to intraoral pain?	
	Do you think you need more knowledge related to psychogenic pain?	

### Pre-test

The questionnaire was pre-tested on 25 participants who were not part of the main study for its comprehension and the average time to fill in. The average time spent in form filling was six minutes. Cron Bach's alpha was applied to measure the internal consistency between responses to the competency questions. A total of 15 items were assessed, and the items were reliable. Cron Bach's alpha for the questionnaire was 0.90. Corrected item-total correlations of the questionnaire ranged from 0.013 to 0.904. An expert health professional assessed the content and face validity of the questionnaire (Asaad JM).

### Ethical aspects

The ethical review committee of Baqai Medical University approved the research protocol for this study (BDC/ERB/2020/001). Informed consent was obtained from the participants.

### Data analysis

Data were analysed using IBM SPSS statistics version 21. Competence items were computed in frequencies and percentages. For the convenience of analysis, responses of a five-point Likert scale related to knowledge, diagnosis and management were recoded and re-categorised into three groups. 1—strongly agree merged with agree, 2—not sure was kept original, 3—strongly disagree was merged with disagree scores. Similarly, 1—no knowledge was merged with limited knowledge, 2—not sure was kept original, 3—extremely knowledgeable was merged with knowledgeable scores, and 1—extremely uncomfortable was merged with uncomfortable, 2—neither was kept original, 3—extremely uncomfortable was merged with comfortable scores. The Chi-square test was conducted to assess the frequency distribution. An independent-sample t-test was used to compare mean scores of knowledge, diagnosis, and management parameters for hypothesis testing. A significant level was set at less than 0.05.

## Results

Of the 475 students, 280 students filled the online survey leaving a response rate of 59%. Total 115 (41.1%) students from public dental schools while 165 (58.9%) students from private dental colleges filled the online survey. Total 152 (54.3%) third-year students, whereas 128 (45.7%) fourth-year students from private and public dental schools filled the online survey. The participants' overall mean age was 22.6 ± 1.95, whereas 56 (20%) were males and 224 (80%) were females.

Dental students perceived degree of knowledge regarding five types of orofacial pain is shown in Table 2. Most dental students, 106 (64%, *p* = 0.016) from private dental schools, feel knowledgeable regarding TMD pain than students of public dental schools. A significant number of fourth-year students, 65 (51%, *p* = 0.005), feels knowledgeable regarding neuropathic pain compared to third-year students. The majority of students from private dental schools, 79 (47.9%, *p* = 0.010), feel they have limited knowledge of psychogenic pain than students at public dental schools.

**Table 2:** The perceived degree of knowledge in the five types of Orofacial pain

Orofacial Pain Type	Variables	Limited knowledge N (%)	Not sure N (%)	Knowledgeable N (%)	*P*-value
TMD pain	Third-year Fourth-year	61 (40) 45 (35)	9 (6) 3 (2)	82 (54) 80 (63)	0.182
	Public sector Private sector	51 (44) 55 (33)	8 (7.0) 4 (3)	56 (49) 106 (64)	0.016
Primary headache	Third-year Fourth-year	67 (44) 51 (40)	22 (15) 9 (7)	63 (41) 68 (53)	0.055
	Public sector Private sector	43 (37) 75 (45)	17 (15) 14 (9)	55 (48) 76 (46)	0.172
Neuropathic pain	Third-year Fourth-year	81 (53) 45 (35)	21 (14) 18 (14)	50 (33) 65 (51)	0.005
	Public sector Private sector	51 (44) 75 (46)	12 (10) 27 (16)	52 (46) 63 (38)	0.280
Intraoral pain	Third-year Fourth-year	50 (33) 32 (25)	8 (5) 4 (3)	94 (62) 92 (72)	0.195
	Public sector Private sector	31 (27) 51 (31)	4 (3) 8 (5)	80 (70) 106 (64)	0.623
Psychogenic pain	Third-year Fourth-year	78 (51) 52 (41)	35 (23) 28 (22)	39 (26) 48 (37)	0.087
	Public sector Private sector	51 (44) 79 (48)	18 (16) 45 (27)	46 (40) 41 (25)	0.010

The majority of third and fourth-year students feel comfortable diagnosing patients with intraoral pain, TMD pain, primary headache, neuropathic pain. On the other hand, most of the students were undecided regarding their comfort level in diagnosing psychogenic pain (Table 3).

**Table 3:** Comfort level related to diagnosing the different types of Orofacial pain

Orofacial Pain Type	Variables	Uncomfortable N (%)	Neither N (%)	Comfortable N (%)	*P*-value
TMD pain	Third-year Fourth-year	27 (18) 11 (9)	26 (27) 28 (22)	99 (65) 89 (69)	0.070
	Public sector Private sector	21 (18) 17 (10)	25 (22) 29 (18)	69 (60) 119 (72)	0.072
Primary headache	Third-year Fourth-year	22 (14) 12 (9.4)	51 (34) 40 (31.3)	79 (52) 76 (59.4)	0.318
	Public sector Private sector	15 (13) 19 (12)	38 (33) 53 (32)	62 (54) 93 (56)	0.895
Neuropathic pain	Third-year Fourth-year	34 (22) 24 (19)	55 (36) 50 (39)	63 (42) 54 (42)	0.740
	Public sector Private sector	25 (22) 33 (20)	42 (36) 63 (38)	48 (41) 69 (42)	0.928
Intraoral pain	Third-year Fourth-year	16 (11) 9 (7)	32 (21) 20 (16)	104 (68) 99 (77)	0.245
	Public sector Private sector	8 (7) 17 (11)	20 (17) 32 (19)	87 (76) 116 (70)	0.532
Psychogenic pain	Third-year Fourth-year	33 (21) 23 (18)	71 (47) 59 (46)	48 (32) 46 (36)	0.642
	Public sector Private sector	20 (17) 36 (22)	50 (44) 80 (48)	45 (39) 49 (30)	0.243

Most of the students from private dental schools, 114 (69%, *p* = 0.009), feel comfortable managing the TMD pain. The majority of the fourth-year students, 100 (78%, *p* = 0.010), feel comfortable managing intraoral pain. Most students were undecided regarding their comfort level in managing psychogenic and neuropathic pain (Table 4).

**Table 4:** Students level of comfort in managing the different types of Orofacial pain

Orofacial Pain Type	Variables	Uncomfortable N (%)	Neither N (%)	Comfortable N (%)	*P*-value
TMD pain	Third-year Fourth-year	23 (15) 10 (8)	42 (28) 29 (23)	87 (57) 89 (69)	0.064
	Public sector Private sector	13 (11) 20 (12)	40 (35) 31 (19)	62 (54) 114 (69)	0.009
Primary headache	Third-year Fourth-year	21 (14) 8 (6)	45 (30) 40 (31)	86 (56) 80 (63)	0.116
	Public sector Private sector	11 (10) 18 (11)	37 (32) 48 (29)	67 (58) 99 (60)	0.833
Neuropathic pain	Third-year Fourth-year	26 (17) 15 (12)	65 (43) 60 (47)	61 (40) 53 (41)	0.434
	Public sector Private sector	14 (12) 27 (16)	54 (47) 71 (43)	47 (41) 67 (41)	0.592
Intraoral pain	Third-year Fourth-year	19 (13) 6 (5)	38 (25) 22 (17)	95 (62) 100 (78)	0.010
	Public sector Private sector	8 (7) 17 (10)	25 (22) 35 (21)	82 (71) 113 (69)	0.626
Psychogenic pain	Third-year Fourth-year	30 (20) 19 (15)	72 (47) 61 (47)	50 (33) 48 (38)	0.503
	Public sector Private sector	21 (18) 28 (17)	58 (50) 75 (45)	36 (32) 62 (38)	0.554

Dental students' perceived need for more knowledge regarding the type of orofacial pain is shown in Table 5. Almost all the students responded that they needed more knowledge related to the five types of OFP.

**Table 5:** Perceived need for more knowledge required regarding the type of orofacial pain

Orofacial Pain Type	Variable	Disagree N (%)	Neutral N (%)	Agree N (%)	*p*-value
TMD pain	Third-year Fourth-year	2 (1) 2 (2)	4 (3) 7 (5)	146 (96) 119 (93)	0.467
	Public sector Private sector	2 (2) 2 (1)	5 (4) 6 (4)	108 (94) 157 (95)	0.891
Primary headache	Third-year Fourth-year	3 (2) 6 (5)	9 (6) 4 (3)	140 (92) 118 (92)	0.251
	Public sector Private sector	1 (1) 8 (5)	5 (4) 8 (5)	109 (95) 149 (90)	0.172
Neuropathic pain	Third-year Fourth-year	0 (0) 1 (1)	6 (4) 4 (3)	146 (96) 123 (96)	0.517
	Public sector Private sector	0 (0) 1 (1)	4 (4) 6 (4)	111 (96) 158 (95)	0.703
Intraoral pain	Third-year Fourth-year	4 (3) 5 (4)	9 (6) 8 (6)	139 (91) 115 (90)	0.826
	Public sector Private sector	4 (4) 5 (3)	7 (6) 10 (6)	104 (90) 150 (91)	0.978
Psychogenic pain	Third-year Fourth-year	2 (1) 1 (1)	3 (2) 4 (3)	147 (97) 123 (96)	0.757
	Public sector Private sector	0 (0) 3 (2)	4 (3) 3 (2)	111 (97) 159 (96)	0.242

Table 6 shows the comparison of knowledge, diagnosis, and management mean scores. Fourth-year students had significantly high mean knowledge (3.19 ± 0.72, *p* = 0.011) and management (3.54 ± 0.48, *p* = 0.011) scores. Comparison of private and public dental schools reveals no significant difference.

**Table 6:** Comparing overall scores of perceived knowledge, diagnosis and management related to OFP types

Variable	Third Year Mean ± S.D	Fourth Year Mean ± S.D	*P*-value	Public Sector Mean ± S.D	Private Sector Mean ± S.D	*P*-value
Knowledge	2.97 ± 0.69	3.19 ± 0.72	0.011	3.09 ± 0.72	3.05 ± 0.71	0.649
Diagnosis	3.36 ± 0.61	3.50 ± 0.55	0.053	3.40 ± 0.63	3.44 ± 0.56	0.663
Management	3.37 ± 0.64	3.54 ± 0.48	0.011	3.43 ± 0.55	3.46 ± 0.59	0.660

## Discussion

To date, studies reporting the perceived competence of OFP among dental students are scarce in Pakistan. The present study was conducted to determine dental students' perceived competence regarding the management of OFP. The understanding of OFP is critical for dental students as they will be at the forefront of the patient management of OFP [14]. It is observed that patients with a complaint of OFP can attend multiple clinicians before seeking or being referred to a dental practitioner with expertise in OFP [14]. Hence the dental students need a good understanding of OFP and require specific training at the undergraduate level.

Chi-square analysis rejects our hypothesis that fourth-year students are more competent in managing orofacial pain. In most OFP categories, no statistically significant difference was found when third- and fourth-year students were compared. However, a statistically significant number of fourth-year students feel more knowledgeable about neuropathic pain and more comfortable managing intraoral pain than third-year students.

Our results showed that third- and fourth-year students of public and private schools reported less knowledge and comfort in diagnosing and managing psychogenic pain. Similarly, Chouchene et al. [15] reported that Tunisian final year students felt uncomfortable managing psychogenic pain. In our study, the students also reported the need for more knowledge related to psychogenic pain. The possible explanation is that students did not get enough didactic exposure and clinical training because psychogenic patients do not commonly visit dental clinics [15, 16].

The fourth-year students of private and public schools reported having more knowledge and feeling more comfortable diagnosing and managing TMD pain and intraoral pain. In addition to that, the frequency of third-year students reported needing more knowledge related to TMD pain and intraoral pain was higher than fourth-year students. This effect may be due to the less clinical exposure of third-year students. Similar results were reported by Alonso et al., 2014 [13]. Similarly, evidence showed that dentist knowledge regarding TMJ disorders increases with a longer duration of practice [17].

Most third-year students reported having limited knowledge and less comfort in diagnosis and managing primary headaches than fourth-year students. That shows that third-year students need more didactic learning of primary headaches. However, fourth-year students reported more knowledge, more comfort in diagnosing and managing primary headaches feel they still need more knowledge related to primary headaches. That may be because they may have been exposed to cases of primary headaches in clinics and realise the complexity of diagnosing and managing primary headaches.

Our study showed that students felt most comfortable diagnosing and managing intraoral pain, followed by TMD pain and other OFP categories. The students feel easy to manage intraoral pain due to the exposure to the dental curriculum and clinical training [13]. On the other hand, students were less comfortable managing TMD pain and other OFP categories because these skills are considered complex and poorly mastered at the end of dental studies when students or recent graduates are interviewed [18–20].

In our study, the frequency of fourth-year students had high mean knowledge scores and high mean scores related to comfort in diagnosing and managing OFP categories than third-year students. Alonso et al. [13] reported similar results when dental students' self-perceived competency at Case Western Reserve University was measured. The possible explanation is that the fourth-year students have more clinical training and didactic learning than third-year students [13].

In addition to that, our results showed that the student reported the need for more knowledge related to OFP categories. This study indicates the need to update the curriculum, teaching methods and incorporate the clinical training related to managing different types of OFP. Evidence shows that short courses for undergraduates on pain with pre and post-test knowledge significantly improved pain knowledge [21, 22]. Similarly, the inclusion of OFP and TMD courses in a semester of third-year curriculum showed improvement in fourth-year dental students' learning at the dental school of Case Western Reserve University [13].

The limitation of the study included a smaller sample size owing to a limited survey response rate. The potential reasons for the lack of participants completing the survey include lack of incentive and survey fatigue due to the rise in survey distribution during the COVID-19 pandemic. Every dental school has different teaching approaches and philosophies. However, the results of this study can be applied to most undergraduate dental students in Karachi, as multiple dental schools participated in this survey. The effects should be assessed carefully as the reported competence is self-perceived, not clinically proven.

## Conclusion

Our hypothesis was rejected in most OFP categories except for intraoral pain. Most fourth-year students feel more comfortable managing intraoral pain and feel more knowledgeable about neuropathic pain than third-year students. In addition, this study found that dental students perceived competence regarding orofacial pain management varies in relation to specific categories, being lowest for psychogenic pain. Diagnosing orofacial pains is highly challenging, and undergraduate dental students feel great impediment to handle the clinical situation. Therefore, for better future clinicians to manage OFP, a multidisciplinary approach during special clinical training sessions of pain management should be implemented at the undergraduate level.

## Data Availability

The datasets used and analysed during the current study are available from the corresponding author upon reasonable request.

## Abbreviations

TMD: Temporomandibular joint disorder
OFP: Orofacial pain
